# Oral Microbiome Research on Oral Lichen Planus: Current Findings and Perspectives

**DOI:** 10.3390/biology11050723

**Published:** 2022-05-09

**Authors:** Won Jung, Sungil Jang

**Affiliations:** 1 Department of Oral Medicine, Institute of Oral Bioscience, School of Dentistry, Jeonbuk National University, Jeonju-si 54907, Korea; jungwon@jbnu.ac.kr; 2 Research Institute of Clinical Medicine, Jeonbuk National University, Jeonju-si 54907, Korea; 3 Biomedical Research Institute, Jeonbuk National University Hospital, Jeonju-si 54907, Korea; 4 Department of Oral Biochemistry, Institute of Oral Bioscience, School of Dentistry, Jeonbuk National University, Jeonju-si 54907, Korea

**Keywords:** oral lichen planus, oral microbiome, oral medicine, oral pathology, buccal mucosa

## Abstract

**Simple Summary:**

Oral lichen planus is a disease of the oral mucosa, which frequently affects women aged 40 years or older. Though the T cell-mediated immune response is involved in the development of oral lichen planus, attempts to identify a microorganism that causes the disease have been unsuccessful. Recent studies on the development of oral lichen planus are focusing on the role of the oral microbiome, which includes oral microbiota and their products, and the host environment. The role of the human microbiome in various diseases has been identified and regulating the microbiome is becoming important in personalized medicine. In this review, we summarized current findings on the role of the oral microbiome in the development of oral lichen planus. The homeostasis of the oral microbiome is disrupted in patients, and functional analysis of oral microbiota and oral mucosa implies that pathways involved in defense against bacterial infection and in the inflammatory response are activated in the oral lichen planus-associated oral microbiome. Though the lack of studies to date makes it difficult to conclude, further studies on the oral microbiome associated with the disease will enable a holistic understanding of the role of the oral microbiome in the development of oral lichen planus and developing a personalized therapy for the disease.

**Abstract:**

Oral lichen planus (OLP) is a chronic inflammatory disease of the oral mucosa with an unknown etiology. The role of oral microbes in the development of OLP has gained researchers’ interest. In this review, we summarized the findings of studies focused on the relationship between OLP and oral microbiome, which includes the composition of oral microbiota, molecules produced by oral microbiota or the host, and the oral environment of the host. According to the studies, the oral microbial community in OLP patients undergoes dysbiosis, and the microbial dysbiosis in OLP patients is more prominent in the buccal mucosa than in the saliva. However, no same microorganisms have been suggested to be associated with OLP in multiple investigations, implying that the functional aspects of the oral microbiota are more important in OLP development than the composition of the oral microbiota. According to studies on host factors that make up the oral environment, signal pathways involved in cellular processes, such as keratinization, inflammation, and T cell responses are triggered in OLP. Studies on the functional aspects of the oral microbiota, as well as interactions between the host and the oral microbiota, are still lacking, and more research is required.

## 1. Introduction

Oral lichen planus (OLP) is a chronic inflammatory disease of the oral mucosa that frequently affects women after the fourth decade of life. OLP can cause pain and discomfort when patients eat, swallow, and speak [1,2]. OLP has been classified into six clinical types: reticular, plaque, atrophic, papular, erosive, and bullous [3]. The most prevalent kinds are reticular (which includes white lines and plaques) and erosive (which includes ulcerations). Erosive and atrophic lesions are often painful, whereas reticular lesions are generally asymptomatic [4]. Although the development of OLP has been linked to a T cell-mediated immune response, the pathogenesis and etiology of OLP remain unexplained. Due to the involvement of immune response during the development of OLP, factors that may trigger an autoimmune response, including oral microbes, have been a subject of research. Many microorganisms, such as *Helicobacter pylori* [5,6], *Mycoplasma salivarium* [7], periodontopathogenic bacteria [8], *Candida albicans* [9,10], human papillomavirus [11], Epstein-Barr virus [12], and hepatitis C virus [13,14,15], have been proposed to be related to OLP development. However, these suggested associations have been denied or remain controversial as there have been other studies that reported opposing results, or mechanisms underlying these associations have been yet identified [16,17,18,19,20,21,22,23]. Moreover, no single microorganism yet fulfills the criteria for a causal relationship with the development of OLP [18,19].

Based on the fact that the human body is colonized by various microorganisms, later studies have been seeking to identify a mechanism of OLP development by focusing on the imbalance of oral microbiota and host response, rather than investigating a single microorganism. In addition to the microorganisms inhabiting the human body, surrounding environmental conditions and products of microbes and the host make up the human microbiome [24]. The human microbiome is being regarded as an important target for diagnosis, prognosis, and treatment of human diseases [25,26], and the effect of gut microbial composition in disease development suggests the possible application of the human microbiome in personalized medicine [27,28]. In this review, we looked into the latest definition of the microbiome and its impact on disease development, summarized the current findings on the association between the oral microbiome and OLP, and discussed future perspectives.

## 2. Microbiome and Human Diseases

Almost all human surfaces that are exposed to the outer environment are occupied with various microorganisms. As the term microbiota refers to all living microorganisms in a defined environment [29], microorganisms colonizing the human body, including skin, gastrointestinal tract, respiratory tract, and urogenital tract, are collectively called human microbiota. Studying microbiota has been difficult due to the fact that many microorganisms that make up the human microbiota are still unculturable [30]. From the beginning of the 21st century, the introduction of massive parallel sequencing (so-called next generation sequencing) technologies enabled studying human microbiota without culturing. Additionally, technical advances have aided studies on human microbiota by lowering the cost of analysis.

Bacteria, archaea, protists, fungi, and viruses make up the human microbiota. Among these, bacteria are thought to constitute the most abundant domain in human microbiota, according to findings from investigations on human gut microbiota [31,32,33]. Regardless of their quantity, every member of the human microbiota serves a role as pathogens or commensals. Interactions among members of the microbiota, as well as interactions between the microbiota and the host, have a variety of effects on the health of the host. For example, *Porphyromonas gingivalis* causes periodontal bone loss by disrupting the balance between commensal microbiota and the host immune response, rather than attacking the bone directly [34]; intestinal commensal bacteria are required for the persistence of pathogenic norovirus infection [35], and interactions with oral bacteria affect *Candida albicans* gene expression and growth [36,37]. These findings highlight the importance of examining the microbial community rather than a single microbial species when investigating probable microbe-related diseases.

The term ‘microbiome’ has been widely used in a variety of domains, including microbiology, ecology, and medicine. However, there has been no clear definition of the term, and it has been used in a variety of ways depending on the context. Recently, a comprehensive definition that covers numerous features of the microbiome has been proposed [24]. According to this, the term ‘microbiome’ encompasses not just microbiota but also molecules produced by microbiota and host, and the environmental condition of the host. The development of analytical tools, such as massive parallel sequencing or mass spectrometry allowed researchers to examine the composition of human microbiota and its products. As a result, progress has been made in the study of the role of the human microbiome in health and disease. The human microbiome has been discovered to have an impact on human physiology, including nutrition, immunity, and development. Furthermore, many chronic diseases are linked to ‘dysbiosis’, or a shift in the composition of the microbiome [38,39]. Commensal gut bacteria, for example, play a role in vitamin synthesis and bile acid metabolism [40]. They also produce metabolites that promote the generation of immune cells in the intestine [41]. Altered gut microbiota affects the onset and progression of diabetes by modulating inflammation and host metabolism [42]. Furthermore, changes in the microbiome have been linked to a variety of disorders.

The oral cavity, like other parts of the human body, is a habitat for a variety of microbes. Even though the oral cavity only makes up a small part of the human body, it is home to a highly diversified microbial community [43,44]. Saliva, dental plaque, and oral mucosa show the highest microbial density in the human body [45,46]. More than 700 bacterial and 100 fungal species are thought to live in the oral cavity [47,48]. A lot of research has investigated the link between the oral microbiome and oral and systemic health. Recent research has linked inflammatory bowel disease to a dysbiosis of the salivary microbiome characterized by an increase in *Bacteroidetes* and a decrease in *Proteobacteria* [49]. Additionally, changes in the oral microbiome have the potential to disrupt the bidirectional link between oral inflammatory disease (periodontitis) and systemic autoimmune diseases, such as rheumatoid arthritis, and systemic lupus erythematosus [50]. Dysbiosis in the oral microbiome is also thought to play a role in the pathogenesis of Alzheimer’s disease [51]. Such associations between the oral microbiome and systemic diseases suggest that the oral microbiome may also be a contributing factor in OLP pathogenesis that has yet to be found.

## 3. Composition of Oral Microbiota in OLP Patients

The relationship between oral microbiota and OLP has mainly been studied by comparing the oral microbial composition of OLP patients and that of healthy controls. Traditionally, unstimulated saliva or buccal swabs have been used to isolate oral microbiota. It has been shown that saliva and buccal mucosa have different bacterial compositions in healthy people: the genera *Neisseria* and *Prevotella* make up roughly half of the salivary bacteria, whereas the genus *Streptococcus* makes up two-thirds of the bacteria in buccal mucosa [52]. Saliva shows a larger bacterial diversity than buccal mucosa, and there is a detectable difference between the two sites [52].

Wang et al. [53] investigated the bacterial makeup and diversity in saliva and mucosal tissue from healthy controls, reticular OLP patients, and erosive OLP patients. Bacterial diversity in OLP patients was higher in saliva than in mucosal tissue, similar to what was found in healthy people [52], and these two sites had distinct community structures [53]. In healthy controls, reticular OLP patients, and erosive OLP patients, the most abundant bacterial genera in saliva were *Neisseria* and *Prevotella*. All three groups showed similar salivary bacterial diversity and community structures [53]. Whereas in buccal tissue, *Curvibacter* and *Halomonas* were the most abundant genera in healthy controls, but these genera were not detected in OLP patients [53]. Though the bacterial diversities in mucosal tissues from healthy controls, reticular OLP patients, and erosive OLP patients were similar, the bacterial community structure of healthy controls could be distinguished from that of OLP patients [53].

Other studies used saliva or buccal swab samples to examine oral microbiota. Yu et al. [54] reported similar alpha diversity in salivary microbial communities from healthy people, erosive OLP, and non-erosive OLP patients. Wang et al. [55] also reported that there was no significant difference in salivary microbial diversity among healthy controls, reticular OLP patients, and erosive OLP patients, but there was a gradual increase in diversity among healthy people to reticular and erosive OLP patients. Erosive OLP patients had a higher number of bacterial taxa in their saliva than healthy controls or reticular OLP patients [55], however, this was not replicated in the study by Yu et al. [54]. According to Zhong et al. [56], the salivary bacterial communities of healthy controls and OLP patients were not significantly different. Li et al. [57], on the other hand, reported that OLP patients had a more diverse salivary bacterial community than healthy controls. However, it has been reported that the salivary bacterial community structure of OLP patients could be distinguished from that of healthy controls [18,54,57].

By analyzing the bacterial composition in the buccal mucosa, Hijazi et al. [58] reported that alpha diversity decreases as the severity of OLP worsens, despite the bacterial diversities in healthy controls and OLP patients not being statistically different. Similar results have been observed in other investigations examining the microbiota in the buccal mucosa, while the significance levels did not meet the pre-defined thresholds. Baek and Choi [18], and He et al. [59] reported that OLP patients had more diverse microbiota in their buccal mucosa than healthy controls. Wang et al. [53], and Du et al. [60] reported that the erosive OLP patients had more diverse microbiota than the reticular OLP patients in the buccal mucosa. Most studies that analyzed the microbial composition in the buccal mucosa have reported that the bacterial community structure of OLP patients can be distinguished from that of healthy controls [53,59,60,61], while one study reported that healthy controls and OLP patients had similar bacterial community structures [58].

Liu et al. [62], on the other hand, studied subgingival bacterial populations in patients with chronic periodontitis with or without gingival erosive OLP. While the bacterial communities of chronic periodontitis patients with gingival erosive OLP were less diverse than those of patients without OLP, the bacterial community structures of the two groups were similar [62]. The genera *Pseudomonas* and *Granulicatella* were shown to be associated with gingival OLP, whereas the genera *Leptotrichia* and *Prevotella* were found to be more abundant in periodontitis patients who did not have gingival OLP [62].

While most research looked at the bacterial community in OLP patients, Li et al. [63] examined the fungal population in the oral cavity of OLP patients using massive parallel sequencing. In comparison to healthy controls, patients with erosive or reticular OLP had a less diverse fungal community in their saliva [63]. The phylum *Ascomycota* was more abundant in OLP patients’ saliva, while the phylum *Basidiomycota* was less abundant [63]. In the saliva of OLP patients, the genus *Aspergillus* was identified more frequently [63]. In each healthy control, reticular OLP, and erosive OLP group, different co-occurrence and co-exclusion patterns were observed when fungal and bacterial populations were examined together [63]. This suggests that the way how bacteria and fungi interact may vary as OLP progresses. Because there are only a few studies on the relationship between the oral fungal community and OLP up to now, more studies on the role of the oral fungal community and bacterial-fungal interaction in the development of OLP are required.

Table 1 summarizes the findings of research that looked at the oral microbial composition in healthy people and OLP patients. There is no agreement among researchers that looked for microbial taxa associated with overall, reticular, or erosive OLP. However, it should be noted that few studies have been conducted to date, and that differences in ethnic groups, diagnostic criteria, and analytic methods may have influenced the findings.

Some studies utilized biopsy tissue to analyze microbial composition [53,64]. According to Wang et al. [53], OLP biopsy tissues displayed similar bacterial diversity to normal tissues, but it should be considered that mucosal tissues obtained during tooth extraction or treatment of sublingual cysts were used as controls. Baek et al. [64] compared the composition of the microbial community in OLP tissues with that on the mucosal surface of OLP lesions. Intratissue bacterial communities were less diverse than those of mucosal surface, and intratissue bacterial structures tended to differ across patients, while those of the mucosal surface tended to cluster together [64]. Among the bacterial species, *Escherichia coli* was found to be significantly enriched in biopsy tissue compared to the buccal mucosa, and *E. coli* strains isolated from OLP tissue were able to invade the oral epithelial cell line and induce apoptosis [64]. Considering that the OLP buccal mucosa shows increased bacterial invasion into the lamina propria and infiltrated T cells [61], analyzing biopsy tissue may be more effective in capturing the OLP-associated microbiota than analyzing mucosal bacterial community obtained by buccal swab.

While there have been many attempts to analyze the composition of the OLP oral microbiota, the effect of microbial load in the oral mucosa has been barely studied. However, it has been reported that plaque control improved subjective and objective symptoms of gingival lichen planus [65], and the amount of plaque and calculus was associated with gingival involvement of OLP [66], suggesting a possible association between the oral bacterial load and OLP.

Overall, the difference in microbial diversity between healthy controls and OLP patients is greater in buccal mucosa than in saliva, suggesting that buccal mucosa may be a better specimen than saliva to study the role of the oral microbiota in the development of OLP. Despite conflicting results, the majority of studies suggest that OLP oral microbiota can be distinguished from healthy oral microbiota. Studies that sought to identify OLP-associated microbial taxa in saliva or buccal mucosa have yielded varying results. Though there is little research to date, mucosal biopsy investigations imply that bacteria invading the mucosa are more closely related to OLP than bacteria in the saliva or on the buccal mucosa [53,61,64]. Despite ethical concerns about harvesting healthy mucosal tissue, current findings highlight the need for more research into the role of intratissue bacteria in the development of OLP.

## 4. Functional Aspects of OLP Oral Microbiota

Sequencing of 16S rRNA gene amplicon has been employed in the majority of microbiome research. This method provides information about the composition of the oral bacterial community, but other domains of oral microbiota, such as fungi and viruses cannot be identified with this method. Moreover, it provides little information about which genes are expressed and how they function. It has been shown that in various microbiome-associated diseases, microbial compositions of diseased people differ from each other, while those of healthy people are alike [67]. Metagenome and metatranscriptome studies on the oral microbiota of periodontitis patients revealed that the microbial composition varies across periodontitis patients, but their microbiomes are functionally congruent, and the difference between healthy and diseased periodontium is better defined by the functional and metabolic activities of microbiome rather than the microbial composition [68,69,70,71]. These imply that the functional characteristics of microbiota, which can be identified by examining transcripts, proteins, and metabolites, should be considered in studying the role of microbiota in disease development.

Until now, few attempts have been made to investigate the functional aspects of the oral microbiota in OLP patients. Li et al. [57] analyzed the salivary microbiome of OLP patients and healthy controls and suggested that genes involved in pathways, such as lipopolysaccharide biosynthesis and lipopolysaccharide biosynthesis proteins are upregulated in the OLP microbiome. Du et al. [60], on the other hand, reported that genes involved in sporulation and ether lipid metabolism were enriched in the buccal mucosa microbiome of OLP patients. However, it should be noted that functional changes reported in these studies were inferred from the results of 16S rRNA gene amplicon sequencing [57,60]. Considering the limitations of prediction based on 16S rRNA gene amplicon sequencing [72], further studies using shotgun metagenome sequencing or RNA sequencing would provide better insights into functional changes in the oral microbiome related to the development of OLP.

Marttila et al. [73] measured the amount of acetaldehyde produced by microbes sampled from oral mucosa. Most microbial samples collected from both healthy controls and OLP patients produced potentially mutagenic levels of acetaldehyde, and no significant difference in acetaldehyde production was observed between healthy controls and OLP patients, or between healthy and affected mucosa of OLP patients [73]. Since the microbial samples were cultured in the lab before being tested for acetaldehyde production [73], it should be considered that the microbial composition might have been changed after the culture. As there have been few functional studies on oral microbiota in OLP patients up to date, more studies focusing on microbial transcription, translation, and metabolism are required to obtain a functional landscape of oral microbiota in OLP patients.

## 5. Changes in Oral Microbiota during Treatment of OLP

Compared to cross-sectional studies comparing the oral microbiome of OLP patients to that of healthy controls, there have been fewer longitudinal studies on changes in oral microbiota during the treatment of OLP. Keller and Kragelund [74,75], and Marlina et al. [76] conducted studies to examine the effect of probiotics on OLP and analyzed how the composition of the oral microbiota changes during treatment. Keller and Kragelund [74,75] treated OLP patients with conventional treatment along with probiotics or placebo, then monitored changes in oral microbial composition during 1 year of treatment and follow-up period. No remarkable changes in the oral microbiome were observed after treatment with topical steroids [75]. Though treatment with antimycotics caused changes in the abundance of some bacterial genera, these changes were restored during the follow-up period after treatment was finished [75]. Overall, the oral microbial composition was barely influenced by conventional treatment or probiotics [75]. Marlina et al. [76] examined the salivary microbial composition and clinical symptoms while treating OLP patients with probiotics or placebo and found that probiotics neither improved clinical symptoms of patients nor changed salivary microbial composition. Results of these studies suggest that probiotics do not alleviate clinical symptoms of OLP [74,76]. However, whether the composition of oral microbiota is related to subjective or objective symptoms of OLP was not investigated in these studies. Further studies addressing these may provide a better understanding of the role of oral microbiota in the development of OLP.

In other studies, changes in the abundance of selected bacterial species during treatment of OLP were examined using quantitative PCR (qPCR) [77,78]. Ku et al. [78] measured changes in the abundance of nine bacterial species associated with dental caries or periodontitis from unstimulated saliva. After being treated with dexamethasone gargle for 4 weeks, patients showed improvement in clinical symptoms of OLP and periodontitis scores [78]. Though most patients showed qualitative changes in more than 1 of the 9 selected bacterial species, no consistent pattern of changes in bacterial species across patients was observed [78]. Cosgarea et al. [77] examined the effect of photodynamic therapy on clinical symptoms of OLP and the abundance of 20 bacterial species in saliva. Four sessions of photodynamic therapy for 2 weeks improved not only objective parameters, such as lesion size and levels of inflammatory cells and cytokines but also the subjective quality of life parameters [77]. However, quantities of selected bacterial species were barely changed after treatment [77]. The fact that the relationship between bacterial species selected in these studies and OLP has not been established, and that changes in the entire oral microbiota cannot be assessed by investigating selected bacterial species, remains a limitation of these studies. Study designs and features of present longitudinal studies on OLP and oral microbiota were summarized in Table 2.

Overall, though there have been only a few longitudinal studies on OLP and oral microbial composition until now, data suggest that the composition of the oral microbiota is unlikely to be affected by conventional or alternative OLP treatment. However, whether the composition of the oral microbiota is associated with treatment outcome is yet to be identified. As demonstrated in various microbiome-associated diseases including periodontitis, functional aspects of the microbiota may be more important than the composition of the microbiota. Based on this, identifying oral microbiota related to treatment outcome by individualized analysis of longitudinal studies may provide a basis for developing a personalized treatment regimen for OLP.

## 6. Changes in Host Factors during Development of OLP

Most microorganisms show specificity to colonize particular hosts or tissues: so-called tropism. OLP-related histopathological changes in mucosal tissue may affect tissue tropism of oral microbiota, resulting in a change in microbial composition. Molecules produced by a host, such as cytokines, metabolites, and other gene expression products, may influence mucosal histology and microbial tropism, and these molecules produced by a host may also be considered components of the microbiome [24].

The expression of inflammatory cytokines in OLP tissues and saliva has long been examined. Increased expression of interleukin (IL)-5, -6, -10, -12, -17, interferon (IFN)-γ, and tumor necrosis factor (TNF)-α has been described in OLP lesions, and increased expression of IL-1, -6, -8, -18, and TNF-α has been described in the saliva of OLP patients [79]. Among these cytokines, IL-1β, IL-8, and TNF-α were reported to be present in higher concentrations in the saliva of erosive OLP patients than those of reticular OLP patients [80]. Moreover, macrophage inflammatory protein (MIP)-1α and -1β, granulocyte macrophage colony stimulating factor (GM-CSF), and IL-6 showed elevated concentration in the saliva of erosive OLP patients compared to healthy controls, while no significant difference was observed between reticular OLP and healthy controls [80].

Studies on oral microbiota revealed an association between changes in cytokine levels and microbial communities. The diversity of microbiota in the buccal mucosa was inversely correlated with the concentrations of IL-1β, IL-10, IL-17α, and IFN-γ in saliva [58]. It has been shown that salivary microbiota and salivary cytokine levels are correlated: the abundances of genera *Alloprevotella* and *Porphyromonas* and the levels of IL-6 and -17 are positively correlated, the abundances of genera *Porphyromonas* and *Fusobacterium* and the levels of IL-6, -8 and -17 are positively correlated, and the abundances of genera *Streptococcus* and *Rothia* and the levels of IL-6, -8 and -17 are negatively correlated [57]. Li et al. [63] found many fungal genera of which relative abundance is positively correlated with salivary IL-17 level and among these, the genera *Erysiphe* and *Bovista* showed a positive correlation with OLP clinical scores.

Changes in metabolite, transcriptomic and proteomic profiles in OLP patients can reflect the change in the metabolic and functional activity and the interaction between microbiota and oral tissue. Differential gene and protein expression, and metabolite production in OLP also have possibilities to be used as diagnostic biomarkers. Vo et al. [81] explored differentially expressed genes (DEGs) in OLP patients using two existing transcriptome datasets: one derived from biopsy tissue (accession no.: GSE38616 [82]), and the other from epithelial layer separated from biopsy using laser microdissection (accession no.: GSE52130 [83]). Overlapping DEGs in the two datasets were associated with epidermal keratinization, wounded oral mucosa, and barrier dysfunction, implying histopathological changes in OLP [81]. Interestingly, genes involved in the response to bacterial infection were upregulated in both datasets [81]. Along with the study conducted by Wang et al. [84], which showed that genes are involved in T cell regulation and inflammation, it can be assumed that interaction between oral mucosa and microbiota takes place during the development of OLP. The transcriptome of total biopsy tissue showed upregulation of genes involved in keratinocyte differentiation and keratinization, while the transcriptome of the epithelial layer showed upregulation of genes involved in immune and inflammatory responses, reflecting differential changes in the mucosal and epithelial layer, respectively [81]. Considering that changes in histopathological characteristics of tissue are associated with dysbiosis in the colonizing microbial community [85,86], the association between changes in gene expression profile in OLP and the oral microbiota can be a topic for further study.

There have been some studies that tried to identify differentially expressed proteins in saliva from OLP patients and healthy controls. Yang et al. [87] pointed out that the expression of urinary prokallikrein is increased, and palate, lung and nasal epithelium clone (PLUNC) is decreased in OLP patients. Talungchit et al. [88] showed the increased expression of fibrinogen fragment D and complement component C3c, and the decreased expression of cystatin SA in OLP patients. Souza et al. [89] focused on the interactions among differentially expressed proteins in OLP patients. Interaction networks of proteins involved in the regulation of inflammation and immune response, cytokine production, and oxidative stress were found in differentially expressed proteins of OLP patients [89]. Overall, findings from proteomics studies suggest that in OLP patients, the expression of proteins associated with the production of inflammatory cytokines, the activity of T lymphocytes, and the response to oxidative stresses are upregulated. Interestingly, an increased concentration of salivary albumin was observed in OLP patients [88,89]. An increase in salivary albumin, which may be caused by leakage of plasma albumin, has been observed in various diseases [90,91,92]. Dysfunction of the epithelial barrier in OLP may cause the increase of salivary albumin, and it is notable that oral bacteria can damage the epithelial barrier [61]. A strong interaction between albumin and lysozyme in OLP patients [89] also implies a role of oral microbiota in the development of OLP. On the other hand, OLP patients showed decreased expression of proteins related to lubrication and viscosity of saliva [89]. This may be related to the fact that OLP is often accompanied by xerostomia [93]. Studies on patients with Sjögren’s syndrome suggest that a decrease in salivary flow affects the composition of oral microbiota [94,95], suggesting that changes in mucosal moistness in OLP may influence the composition of oral microbiota.

Metabolites from saliva or serum have been used to identify differential metabolic pathways between OLP patients and healthy controls. Though there have been few studies until now, metabolite analyses on saliva and serum showed overlapping results. Metabolic pathways, such as D-glutamine and D-glutamate metabolism, alanine, aspartate and glutamate metabolism, and arginine and proline metabolism were found to be disturbed in OLP patients [96,97,98,99]. These metabolic pathways were further dysregulated in oral squamous cell carcinoma compared to OLP [100,101], probably supporting that OLP is a precancerous lesion.

One phenomenon which may affect the homeostasis of host immune response is allergy. Chronic allergic inflammation results not only in the infiltration of immune cells but also in changes in the extracellular matrix and tissue functions [102], which may affect the tissue tropism of oral microorganisms. Dental materials used for restoration or prosthetics exist in the oral cavity for the long term and sometimes evoke an allergic reaction. It has been reported that allergy can cause changes in the oral microbiome [103,104], and some studies suggest a possible association between allergy and the development of OLP [105,106,107]. Along with the difference in allergy prevalence across sex [108], the sex-associated differences in the oral microbiome [109,110,111] may contribute to the biased distribution of OLP in aged women.

Changes in the oral environment, including gene and protein expression, and metabolite production of the oral mucosa, may be a response to microbial dysbiosis. On the other hand, changes in the oral environment can also affect the composition and function of the oral microbiota. The way how the oral environment of the host and the oral microbiota interacts requires further investigation.

## 7. Conclusions and Perspective

Before the widespread adoption of massive parallel sequencing technology, the association between oral microorganisms and OLP has been studied using targeted approaches, such as PCR, culture, or serological analysis. By utilizing these methods, various microorganisms have been suggested as causative agents of OLP. Despite the fact there has not been much research examining the oral microbiota in OLP patients until now, relationships suggested by previous studies with targeted approaches are unlikely to be supported by results from recent microbiome analyses. The majority of the oral microbiome research focused on the oral bacterial community, while other members of oral microbiota, such as fungi or viruses have been paid less attention. Based on currently available studies, no specific microorganism can be pointed to as the causative agent of OLP as each study reports distinct microbial taxa to be associated with the disease. The insufficiency of data available so far, especially on fungi or viruses, would be supplemented by further investigations. Though studies seeking to find out the microbial taxa associated with OLP have reported conflicting results, it appears to be evident that the oral microbiota of OLP patients undergo dysbiosis. Studies suggest that dysbiosis in the oral microbiota is more prominent in erosive OLP than in reticular OLP, and it can be more easily observed in buccal mucosa than in saliva. Inconsistent results found in multiple studies on the oral microbial composition of OLP patients imply that functional changes in the oral microbiota of OLP patients should be questioned, rather than the composition of the oral microbiota itself. In addition, it would be meaningful to focus on the change in the oral microbiota of OLP-affected individuals using longitudinal studies. Monitoring the changes in the oral microbiota during treatment of OLP along with the treatment outcome may help to identify microbial factors related to treatment response. Changes in the oral environment during the development of OLP have been well studied, and results suggest that cellular pathways related to keratinization, inflammation, and T cell responses are activated. A disturbance of such cellular pathways according to alteration of the oral microbiome is also associated with the development of oral cancers. Some bacterial species are linked to both premalignant lesions and oral squamous cell carcinoma (OSCC) [112]. Cellular invasion or oral bacteria may induce the development of OSCC through the overexpression and upregulation of cell signaling [113,114]. This suggests that dysbiosis of the oral microbiome could play an important role in the malignant transformation of OLP. Thus, alteration in the oral microbiome may be able to be used as a predictive marker for malignant transformation [115]. However, the effect of dysbiosis in the oral microbiome during the malignant transformation of OLP is yet unknown. Further research is required to identify how the oral microbiota interacts with the oral environment during the development and progression of OLP. To conclude, in addition to analyzing the difference between the oral microbial composition between healthy people and OLP patients, further studies on the functional characteristics of the OLP oral microbiome, the interaction between the oral microbiota and host environment, and the changes in oral microbiome during treatment of OLP would improve a holistic understanding of the etiology of OLP, and provide basic knowledge for developing personalized treatments for OLP.

## Figures and Tables

**Table 1:** Oral microbial genera reported to be associated with OLP in studies utilized massive parallel sequencing technology.

Study [Reference]	Method	Sample	Group (N)	Related Genera
Wang et al., 2016 [55]	16S rRNA sequencing	unstimulated saliva	healthy control (18)	*Haemophilus*, *Corynebacterium*, *Cellulosimicrobium*, *Campylobacter*
			reticular OLP (19)	*Solobacterium*
			erosive OLP (18)	*Porphyromonas*
Choi et al., 2016 [61]	16S rRNA sequencing	buccal mucosa	healthy control (11)	*Streptococcus*, *Escherichia*
			OLP (13)	*Leptotrichia*, *Acinetobacter*
He et al., 2017 [59]	16S rRNA sequencing	buccal mucosa	healthy control (21)	*Actinobacillus*
			OLP (43) ^a^	*Actinomyces*, *Veillonella*, *Lautrophia*, *Leptotrichia*
Li et al., 2019 [63]	ITS2 region sequencing	unstimulated saliva	healthy control (18)	*Ascomycota_unidentified_1_1*, *Trichosporon*
			reticular OLP (17)	*Candida*, *Aspergillus*
			erosive OLP (18)	*Alternaria*, *Sclerotiniaceae_unidentified*
Du et al., 2020 [60]	16S rRNA sequencing	buccal mucosa	healthy control (10)	*Streptococcus*, *Neisseria*
			OLP (20) ^b^	*Fusobacterium*, *Granulicatella*
Baek et al., 2020 [64]	16S rRNA sequencing	buccal mucosa	OLP (7)	*Haemophilus*, *Neisseria*, *Fusobacterium*
		biopsy tissue	OLP (7)	*Escherichia*, *Acinetobacter*, *Sphingomonas*
Yu et al., 2020 [54]	16S rRNA sequencing	unstimulated saliva	healthy control (10)	*Abiotrophia*, *Eikenella*, *Aggregatibacter*, *Bacteroides*, *Neisseria*, *Ezakiella*
			non-erosive OLP (10)	*Haemophilus*, *Oribacterium*
		data	erosive OLP (10)	*Rothia*, *Oribacterium*
Wang et al., 2020 [53]	16S rRNA sequencing	unstimulated saliva	healthy control (20)	ND
			OLP (40) ^c^	*Capnocytophaga*, *Gemella*, *Granulicatella*
		biopsy tissue	healthy control (4)	*Streptococcus*, *Micrococcus*, *Sphingobium*
			OLP (20) ^d^	*Escherichia-Shigella*, *Phyllobacterium*, *Megaspaera*
Hijazi et al., 2020 [58]	16S rRNA sequencing	buccal mucosa	healthy control (13)	ND
		data	OLP (18)	ND
Zhong et al., 2020 [56]	RNA sequencing	unstimulated saliva	healthy control (5)	ND
		data	OLP (10)	ND
Li et al., 2021 [57]	16S rRNA sequencing	unstimulated saliva	healthy control (21)	*Streptococcus*, *Rothia*
			OLP (30)	*Prevotella*, *Alloprevotella*, *Fusobacterium*, *Porphyromonas*
Liu et al., 2021 [62]	16S rRNA sequencing	subgingival plaque	chronic periodontitis (20)	*Leptotrichia*, *Prevotella*
			chronic periodontitis with gingival erosive OLP (19)	*Pseudomonas*, *Granulicatella*

^a^ non-erosive OLP (22) and erosive OLP (21) grouped together. ^b^ reticular OLP (10) and erosive OLP (10) grouped together. ^c^ reticular OLP (20) and erosive OLP (20) grouped together. ^d^ reticular OLP (12) and erosive OLP (8) grouped together. ND. not described.

**Table 2:** Longitudinal studies on changes in oral microbiota during treatment of OLP.

Study [Reference]	Method	Sample	Intervention	Description
Keller and Kragelund, 2018 [74], Kragelund and Keller, 2019 [75]	16S rRNA sequencing, ITS1 region sequencing	mouthwash, buccal mucosa	conventional treatment (antimycotics or steroids) with or without probiotics	Total number of participants: 22 Patients were followed up until 1 year from the beginning of the study
Cosgarea et al., 2020 [77]	qPCR	unstimulated saliva	photodynamic therapy	Total number of participants: 20 Number of bacterial species analyzed: 20
Ku et al., 2021 [78]	qPCR	unstimulated saliva	dexamethasone gargle	Total number of participants: 20 Number of bacterial species analyzed: 9
Marlina et al., 2021 [76]	16S rRNA sequencing	unstimulated saliva	probiotics	Total number of participants: 27 Administration of topical medications (analgesics, corticosteroids, immunosuppressants) were not controlled

## Data Availability

Not applicable.
